# Case report: STRN3-NTRK3 fusion in uterine sarcoma with spleen metastasis: a new variant in the spectrum of NTRK-rearranged tumors

**DOI:** 10.3389/fmed.2024.1448491

**Published:** 2024-11-08

**Authors:** Piergiuseppe Colombo, Giuseppina Adriana Buonamassa, Anita Giulianini, Letizia Hassan, Noemi Rudini, Antonio Rizzo, Enrico Cavallo, Carlo Carnaghi, Salumeh Goudarzi, Sebastiano Mongiovì, Sarah Pafumi, Stefano Marletta

**Affiliations:** ^1^Department of Biomedical Science, Humanitas University, Milan, Italy; ^2^Department of Pathology, IRCCS Humanitas Research Hospital, Rozzano, Milan, Italy; ^3^Department of Pathology, Humanitas Istituto Clinico Catanese, Catania, Italy; ^4^Department of Medical Oncology, Humanitas Istituto Clinico Catanese, Catania, Italy; ^5^Department of Radiology, Humanitas Istituto Clinico Catanese, Catania, Italy; ^6^Department of Surgery, Humanitas Istituto Clinico Catanese, Catania, Italy; ^7^Section of Oncology, Department of Medicine, University of Verona, Verona University Hospital Trust (AUOI), Verona, Italy; ^8^Section of Pathology, Department of Diagnostics and Public Health, University of Verona, Verona, Italy

**Keywords:** neurotrophic tyrosine receptor kinase (NTRK), uterine sarcoma, STRN3-NTRK3, rearranged tumors, targeted therapy

## Abstract

Neurotrophic tyrosine receptor kinase (NTRK) fusions are infrequent genetic events that can occur in various tumor types. Specifically, NTRK-rearranged sarcoma has been observed in pediatric mesenchymal tumors and, to a lesser extent, in adult mesenchymal tumors like fibrosarcoma. Recently, NTRK-rearranged uterine sarcoma (US) has been identified as a rare entity characterized by constitutive activation or overexpression of the TRK receptor, which plays a role in cell proliferation and differentiation. Since its initial description in 2018, only 46 cases of NTRK-rearranged US have been reported. In this context, herein we describe an exceptional case of an *STRN3::NTRK3* fused US with histologically confirmed splenic metastasis. Notably, such localization has not been previously associated with pure uterine sarcomas in the literature. The fusion involved *STRN3* (exon-3) and *NTRK3* (exon-14) genes and was identified through next-generation sequencing analysis. Recognizing this specific molecular rearrangement is crucial, as it not only enables targeted therapy but also holds diagnostic significance in specific clinical scenarios.

## 1 Introduction

*NTRK* gene rearrangements are driver molecular alterations found in a wide variety of human cancers, including both common and rare tumors such as infantile fibrosarcomas, lipofibromatosis-like neural tumors, and fibrosarcoma-like uterine sarcomas (1). NTRK-rearranged uterine sarcomas are rare malignant spindle-cell neoplasms that typically arise in the uterine cervix, uterine corpus, and vagina. They primarily affect premenopausal women, with an average age of 35 years (median 30.5, ranging from 23 to 60 years) (2). Since the first report by Chiang et al. (3), only 46 cases of these rare tumors have been described. Despite their morphologically unremarkable appearance and often organ-confined nature at initial diagnosis, these neoplasms generally display aggressive clinical behavior, characterized by rapid recurrence and metastatic dissemination (4). Even though a limited number of cases has been described to date, a recent review has identified the presence of either coagulative necrosis, high mitotic count (≥8/10 HPF), lymphovascular invasion, or *NTRK3* rearrangements as independent prognostic features predicting a high-risk of recurrence/metastases (5).

Tumors with *NTRK* gene alterations can benefit from TRK inhibitor-targeted therapy, especially in advanced or metastatic disease. Proper classification of the lesion and identification of these pathogenic mutations are clinically significant. For instance, Michal et al. (6) observed that an *STRN::NTRK3* fusion in a mesenchymal tumor of the uterus was characterized by bland morphology and subsequently defined as low-grade unclassifiable. In this context, we report the first case of *STRN3::NTRK3* fused high-grade sarcoma of the uterus with histologically documented splenic metastasis. This finding could expand the spectrum of these rare and fascinating entities and may be further clarified by WHO classification in the future.

## 2 Case description

**Figure 1:**
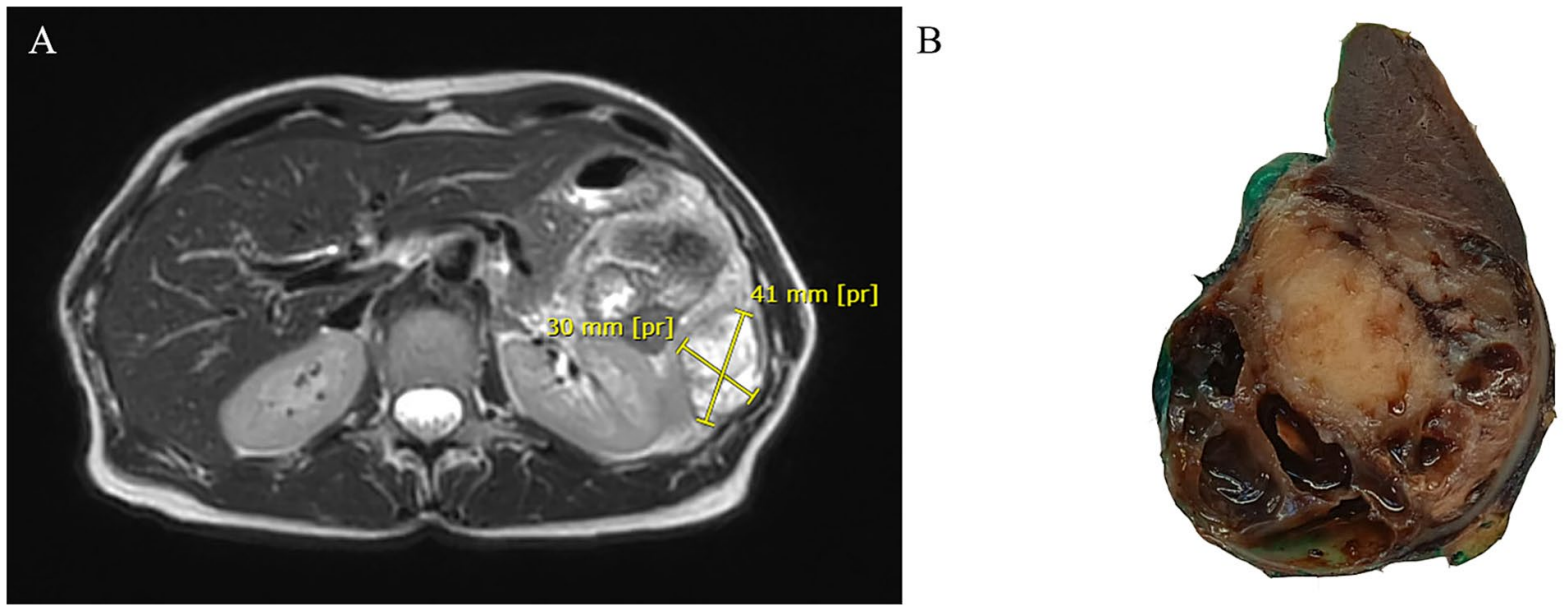
Abdominal MRI showing a cystic mass in the inferior pole of the spleen with peripheral enhancement in T2-weighted sequences (A). Gross section of the surgical specimen revealing a roundish mass with pushing borders at the lower pole of the spleen alternating solid and cystic areas (B).

A 58-year-old woman presented with left-sided flank abdominal pain. In her clinical history, she reported five previous pregnancies and had undergone 6 years before bilateral radical hysterectomy with bilateral uterine tube and ovary resection for a uterine polypoid neoplasm. Such a tumor was made up of a highly cellular proliferation of atypical spindle and, at times, roundish cells with a high mitotic index displacing normal uterine glands. Therefore, it had been diagnosed as an undifferentiated uterine sarcoma with “adenosarcoma-like features” due to peripheral entrapment of benign cervical glands. Abdominal ultrasound revealed a splenic cystic mass, which was subsequently confirmed by CT scan and MRI to be a 30 × 41 mm complex solid-cystic mass in the inferior pole of the spleen, showing peripheral enhancement (Figure 1A). Left para-aortic lymphadenopathy was also noted. Following a multidisciplinary consultation, the patient underwent radical splenectomy.

**Figure 2:**
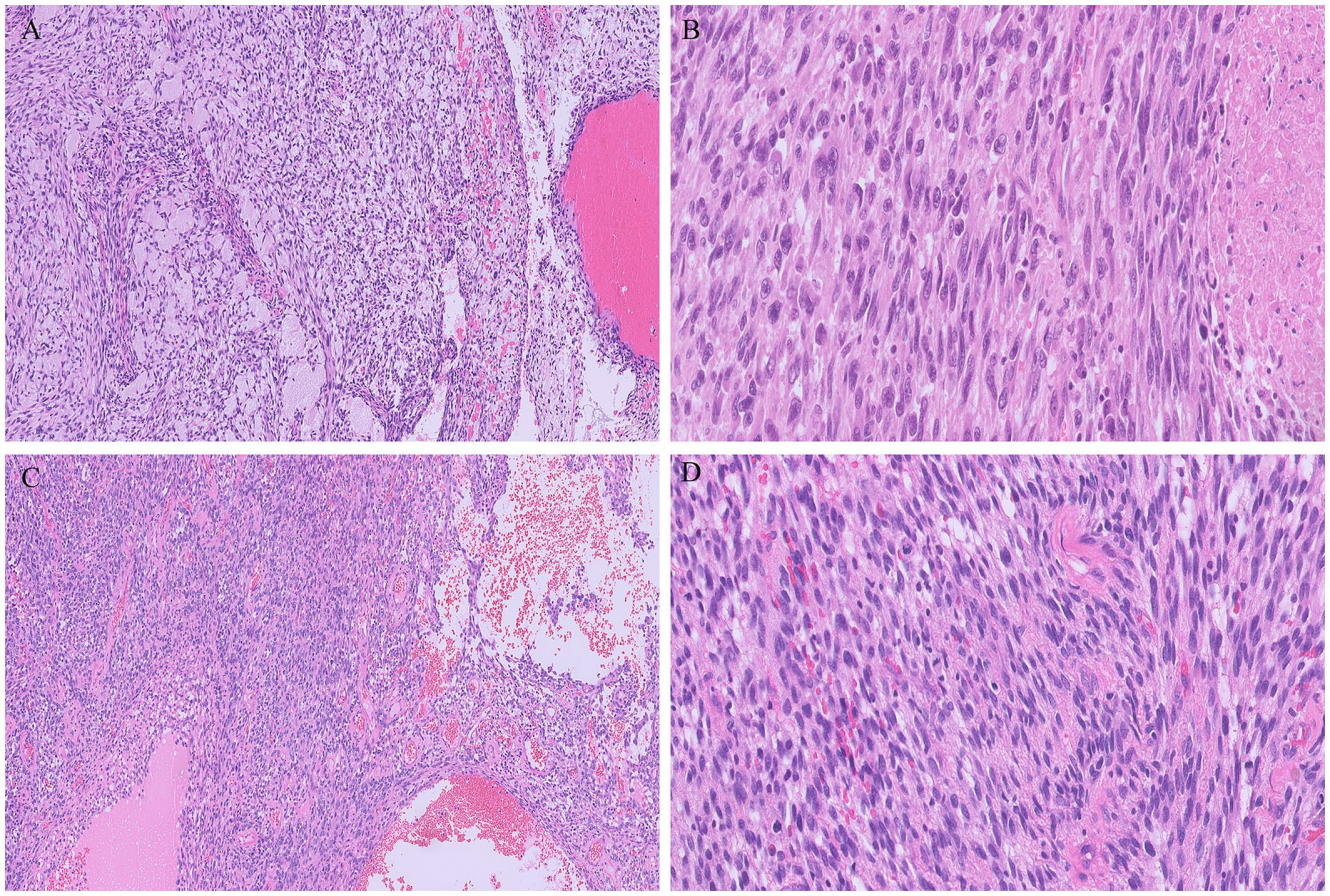
Metastatic tumor deposit in the spleen consisting of solid proliferation of spindle at times with a herringbone pattern of growth, intermixed with cystic areas, in a myxoid stroma (A). Multiple foci of necrosis and pleomorphic cells were evident throughout the tumor (B). The metastatic tumor morphologically resembled the uterine neoplasm (C,D), without characteristic architectural growth nor cellular differentiation reminiscent of a specific lineage.

**Figure 3:**
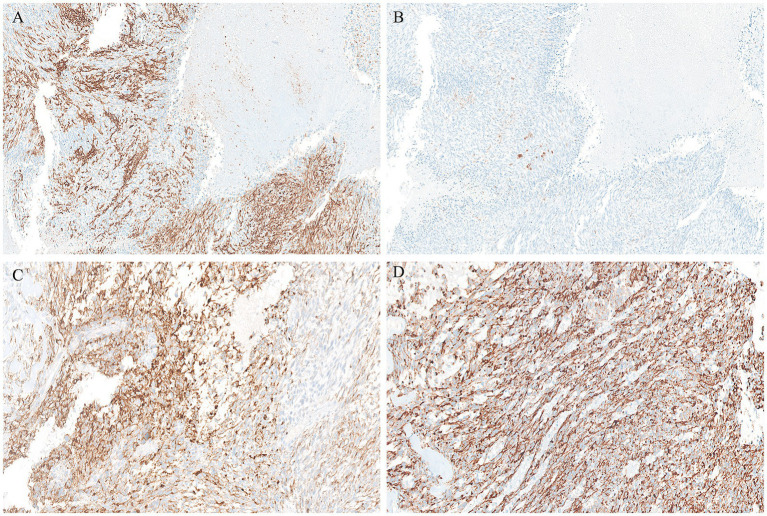
Positive CD34 immunohistochemical staining in the splenic metastasis (A). Conversely, S100 labelled only a few scattered cells (B). Both the splenic localization (C) and the primary uterine sarcoma (D) displayed strong PanTRK cytoplasmic expression.

**Figure 4:**
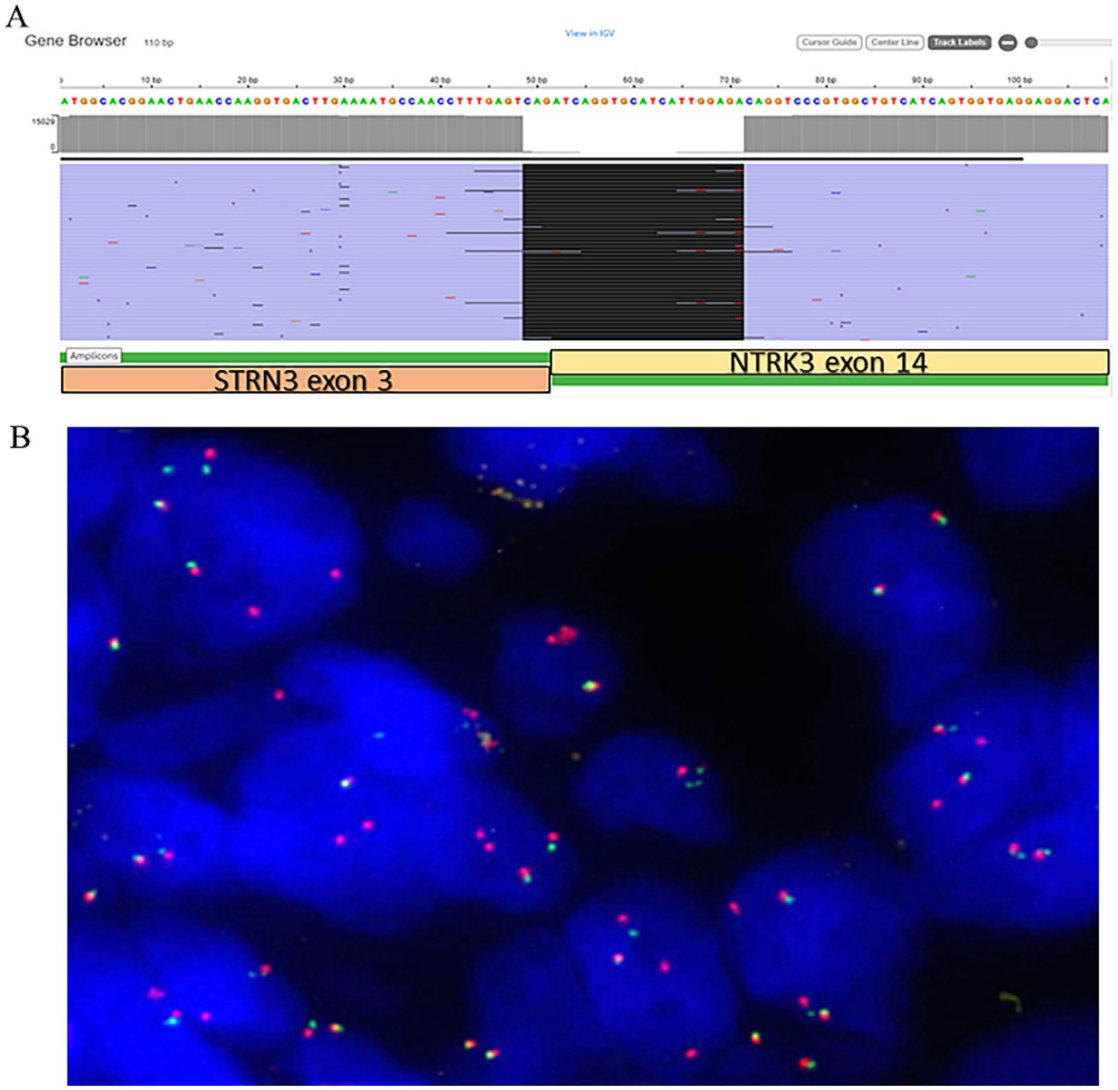
(A) Schematic presentation of *STRN3::NTRK3* not targeted fusion in the current case (view in IGV) with the breakpoint sequence indicated. (B) *NTRK3* dual color break apart FISH probes showing fused unbroken probe (yellow signals), broken 5′and 3′ probes (separated red and green signals) and multiple extra 3′ red signals in 90% of tumor cells.

Gross examination of the spleen revealed an irregular and multinodular appearance due to a 4.5 × 4 × 4 cm roundish intraparenchymal lesion. This lesion exhibited mucoid-myxoid, cystic-hemorrhagic, and solid gray-yellowish areas on the cut surface (Figure 1B). Paraffin-embedded formalin-fixed blocks were cut into 4 μm slides and stained with H&E. Immunohistochemistry was performed on 4 μm sections using various primary monoclonal antibodies. Notably, pan-TRK immunostaining was also conducted to analyze *NTRK* gene alterations. Morphologically, the neoplasm was made up of a solid-arranged proliferation of spindle and pleomorphic cells with pushing margins, along with cystic areas and myxoid stroma (Figure 2A). Geographic necrosis was observed throughout the tumor, coupled with a high mitotic rate (18/10HPF) (Figure 2B). Despite lacking characteristic architectural growth or differentiation reminiscent of a specific lineage, the histological features, combined with the patient’s previous history of a uterine polypoid neoplasm, led to the hypothesis of splenic localization by the primary uterine sarcoma (Figures 2C,D). The neoplastic cells of both the previously removed uterine tumor and the novel splenic localization exhibited the following immunophenotype: WT1+, CD10+, CD34+/− (Figure 3A), S100+ (Figure 3B), Smooth Muscle Actin+/−, ERG−, Desmin−, HMB45−, PanCK−, CK7−, p16−, ER−, PR+ (5%), AR−, AFP−, Myogenin−, and MyoD1−. The proliferation index (Ki-67) was 40%. Based on these findings, a diagnosis of high-grade spindle and pleomorphic cell sarcoma NOS (grade III FNCLCC—D3N1M2) was made. Pan-TRK immunostaining was positive in the majority of the neoplastic cells both in the splenic metastasis and primary uterine neoplasm (Figures 3C,D). To confirm pan-TRK immunopositivity, NGS-based fusion detection analysis was performed. RNA was purified, and sequencing revealed a fusion of *STRN3* (exon 3)—*NTRK3* (exon 14) genes (15,000 read count) in the splenic tumor, which was later also detected in the uterine neoplasm (Figure 4A). Further validation was achieved through fluorescence *in situ* hybridization (FISH) using a Dual Color Break Apart Probe (targeting the *NTRK3* gene at 15q25.3) on tissue from the splenic metastasis, which confirmed the presence of an *NTRK3* rearrangement (Figure 4B).

Ultimately, the diagnosis of splenic metastatic TRK-rearranged sarcoma originating from a uterine primary was established. As for the present case, according to the previously mentioned prognostic score (5), it could be labeled as a high-risk tumor, due to the identification of three risk factors (high mitotic count, coagulative necrosis, *NTRK3* rearrangement). Six months after surgery, two small PET-positive nodules in the Morrison peritoneal zone were noticed. Therefore, after multidisciplinary discussion, the patient was considered suitable for anti-NTRK target therapy (larotrectinib), which she is about to start in the following weeks.

## 3 Discussion

The most common malignant mesenchymal neoplasm of the uterus is leiomyosarcoma, followed by low/high-grade endometrial stromal sarcoma and undifferentiated sarcoma. The WHO classification defining the different categories of uterine sarcomas is becoming increasingly complex due to the continuous discovery of cases associated with new rare mutations (7). Uterine sarcomas with *NTRK* gene rearrangements constitute a recently identified group of uterine spindle-shaped cell tumors, which were likely previously grouped under the spectrum of undifferentiated uterine sarcoma. These NTRK-rearranged sarcomas have the potential to respond to TRK inhibitors (2). The first case was reported by Chiang et al. (3), and to date, only 46 cases have been described in the literature (4, 6–8). Most of these tumors have been identified due to the expanding accessibility of advanced molecular methodologies, such as next-generation sequencing (NGS), which has unveiled novel diagnostic molecular alterations.

*NTRK* rearrangements are oncogenic events occurring in a wide variety of human cancers, including both common and rare tumor types such as infantile fibrosarcomas, lipofibromatosis-like neural tumors, and fibrosarcoma-like uterine sarcomas (1). The *NTRK* gene family encodes the TrkA, TrkB, and TrkC proteins, which activate downstream signaling cascades via PI3K, RAS/MAPK/ERK, and PLC-gamma pathways, ultimately leading to cellular proliferation (1).

In this report, we present the first case of *STRN3::NTRK3* fused uterine sarcoma metastatic to the spleen, properly classified after the excision of the secondary distant localization. The rarity of this case lies in the significant impact that the diagnosis has on the patient’s management, with the prospect of treating potential future metastases using NTRK inhibitors. Currently, this specific variant of rearranged sarcoma is included in the WHO classification as ‘NTRK-rearranged spindle cell neoplasms’ (NTRK-SCN). This category encompasses spindle cell tumors occurring in both children and adults, forming a continuous morphological spectrum (9). The morphological features of our case align with what has been reported in the literature: the tumor consisted of poorly organized spindle and pleomorphic cells, displaying an infiltrative growth pattern within a background of myxoid and cystic stroma. Hypercellular areas, hemorrhagic foci, and geographic coagulative necrosis were also observed. As frequently noticed in other similar reports, the neoplastic cells entrapped normal uterine glands, giving the tumor an “adenosarcoma-like” appearance. However, the lack of a clear-cut peripheral cuffing of native glands by neoplastic cells is a useful morphological hint to avoid misdiagnoses (10, 11). Across the morphological spectrum of NTRK-SCN, many tumors exhibit variable immunohistochemical co-expression of CD34 and S100 protein (12, 13). Their molecular backgrounds involve various gene fusions and, less frequently, point mutations in kinase genes such as *NTRK1/2/3* (1). Similarly, our case showed focal positivity for both CD34 and S100, while additional immunoreactivity may be present but is less specific for this type of neoplasm (e.g., WT1, CD10, desmin, and smooth muscle actin; not shown).

Given that these neoplasms may exhibit histological overlap with other entities, further investigation is crucial to avoid misdiagnoses. Consequently, we decided to perform pan-TRK immunohistochemical testing, which revealed expression in 50% of the cellular population. Although pan-TRK expression lacks absolute sensitivity or specificity for NTRK-rearranged sarcomas, these neoplasms typically exhibit diffuse staining of moderate to strong intensity, unlike their mimics (14). Ultimately, molecular confirmation by NGS was necessary for the diagnosis, as it outperforms other tests such as FISH or immunohistochemistry. An advantage of NGS is its ability to identify multiple oncogenic events alongside *NTRK* gene fusions from a single tumor sample, providing comprehensive insights into the molecular landscape of the investigated neoplasm (15). In our case, NGS results yielded interesting findings: the assay revealed an *STRN3::NTRK3* fusion, with breakpoints involving exon 3 of the *STRN3* gene and exon 14 of the *NTRK3* gene. Regarding uterine tumors, this specific fusion has been previously reported only once by Michal et al. (6) (Table 1). Notably, although the tumor described by Michal et al. (6) was positive for S100 and CD34, with pan-TRK expression, its histological features differed from ours. Our findings align more closely with what Yamazaki et al. (16) recently described for adult fibrosarcoma, expanding the spectrum of NTRK-SCN rearrangements in the uterus. To date, *STRN::NTRK* translocations are rare, and the occurrence of the *STRN3* variant is even rarer (Table 1) (17–20). The *STRN3* gene, mapped at chromosome 14q12, encodes for the striatin 3 protein (also called SG2NA). Along with the other proteins of the striatin family, codified by the *STRN* and *STRN4* genes, it acts as a calmodulin-binding protein physiologically playing a relevant role in estrogen signaling and neuroprotection (21). Rearrangements of the striatin family genes have been claimed as putative molecular drivers of different pathological conditions, including arrhythmogenic cardiomyopathies and cancer (21). As for this latter, apart from the above mentioned *STRN::NTRK* fusions in adult fibrosarcomas, *STRN::ALK* fusions have been identified in some thyroid and lung carcinomas, where they are thought to lead to ligand-independent activation of ALK kinase (16).

**Table 1:** Described *STRN::NTRK* fused cases stratified by subtype, organ, grading, and specific fusion pair.

Reference	Subtype	Primary site	No. cases	Sex	Age	Size (cm)	Grading	Fusion pair	Method	FU (mo)
Lin et al. (17)	UPS	Gluteal region	1	F	35	16.5	High	*STRN::NTRK2*	RNA-based NGS	DOD
Boyer et al. (18)	Glioneuronal tumor	CNS	1	M	53	N/A	High	*STRN1::NTRK2*	RNA-based NGS	NED, 16
Gao et al. (19)	Sarcoma NOS	Duodenum	1	M	63	5	High	*STRN::NTRK2*	RNA-based NGS	NED, 30
Alvarez-Breckenridge et al. (20)	Ganglioglioma	CNS	1	N/A	33	N/A	WHO I	*STRN3::NTRK2*	Multiplex PCR	N/A
Kubota et al. (22)	Fibrosarcoma	Right thigh	1	M	26	11.5	Intermediate	*STRN::NTRK3*	RNA-based NGS	DOD, 29
	Fibrosarcoma	Left radius	1	F	38	8.7	Low	*STRN3::NTRK3*		AWD, 113
Michal et al. (6)	Uterine sarcoma	Uterus	1	F	26	23	Benign	*STRN3::NTRK3*	RNA-based NGS	NED, 36
Present case	Uterine sarcoma	Uterus	1	F	58	4	High	*STRN3::NTRK3*	RNA-based NGS	AWD, 6

FU: follow up; UPS: undifferentiated pleomorphic sarcoma; AWD, alive without the disease; CNS, central nervous system; DOD, died of disease; F, female; M, male; N/A, not available; NED, no evidence of disease; NOS, not otherwise specified; NGS, next-generation sequencing; NTRK, neurotrophic tyrosine receptor kinase; PCR, polymerase chain reaction.

The clinical significance of the exceptional genetic finding in our case lies in its impact on patient treatment. Tumors harboring *NTRK* rearrangements can potentially benefit from targeted therapy with TRK inhibitors (such as entrectinib and larotrectinib, approved in 2020 and 2021, respectively), which emerge as highly effective options in the event of potential metastases (22). Given the rarity of these tumors, further research is needed to better understand optimal management strategies and long-term outcomes associated with NTRK-rearranged uterine sarcomas. In localized diseases, surgical resection through hysterectomy remains the first-line treatment. Systematic genetic analysis is essential, especially for patients at high risk of relapse or in the recurrence setting, as sarcomas associated with *NTRK* fusion transcripts can benefit from targeted therapy (8). Current guidelines from the French Sarcoma Group and the Rare Gynecological Tumors Group emphasize integrating molecular data with accurate histopathological findings to enhance the identification of NTRK-rearranged uterine sarcomas, potentially improving clinical outcomes and enhancing patients’ quality of life (23).

This rare case underscores the importance of recognizing *NTRK* rearrangements, not only for treatment but also for diagnostic purposes in high-risk uterine sarcomas. Close collaboration among multidisciplinary teams remains crucial for comprehensive care in patients with NTRK-rearranged uterine sarcomas.

## Data Availability

The original contributions presented in this study are included in the article, further inquiries can be directed to the corresponding author.
